# The importance of glycogen molecular structure for blood glucose control

**DOI:** 10.1016/j.isci.2020.101953

**Published:** 2020-12-16

**Authors:** Asad Nawaz, Peng Zhang, Enpeng Li, Robert G. Gilbert, Mitchell A. Sullivan

**Affiliations:** 1 Jiangsu Key Laboratory of Crop Genetics and Physiology, Key Laboratory of Plant Functional Genomics of the Ministry of Education, College of Agriculture, Yangzhou University, Yangzhou 225009, P.R. China; 2 Co-Innovation Center for Modern Production Technology of Grain Crops, Yangzhou University, Yangzhou 225009, China; 3 School of Electronic Information Engineering, Yangtze Normal University, Chongqing, 408100, China; 4 The University of Queensland, Centre for Nutrition and Food Sciences, Queensland Alliance for Agriculture and Food Innovation, Brisbane 4072, QLD, Australia; 5 Glycation and Diabetes, Mater Research Institute – The University of Queensland, Translational Research Institute, Brisbane, QLD 4102, Australia

**Keywords:** Glycobiology, Molecular Structure, Diabetology

## Abstract

Type 2 diabetes incidence continues to increase rapidly. This disease is characterized by a breakdown in blood glucose homeostasis. The impairment of glycemic control is linked to the structure of glycogen, a highly branched glucose polymer. Liver glycogen, a major controller of blood sugar, comprises small β particles which can link together to form larger α particles. These degrade to glucose more slowly than β particles, enabling a controlled release of blood glucose. The α particles in diabetic mice are however easily broken down into β particles, which degrade more quickly. Because this may lead to higher blood glucose, understanding this diabetes-associated breakdown of α-particle molecular structure may help in the development of diabetes therapeutics. We review the extraction of liver glycogen, its molecular structure, and how this structure is affected by diabetes and then use this knowledge to make postulates to guide the development of strategies to help mitigate type 2 diabetes.

## Highlights

• Diabetes involves uncontrolled blood glucose levels
• Liver glycogen acts as a blood glucose buffer
• Diabetes can lead to molecularly fragile liver glycogen particles
• Molecularly fragile liver glycogen may exacerbate poor blood glucose control

## Graphical Abstract

**Figure:**
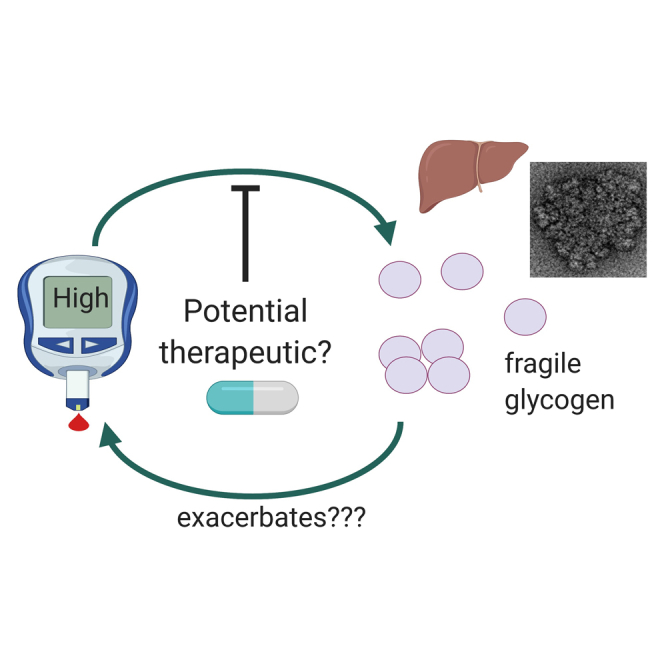

## Introduction

Diabetes has become an increasingly pressing global concern, with the incidence growing rapidly over recent decades in both developed and developing countries, probably due to changes in lifestyles. It is estimated that the incidence of diabetes has risen from 151 million people in 2000 to 463 million in 2019, with 4.2 million estimated deaths in 2019 (International Diabetes Federation, 2019), and that over 700 million people will be affected with diabetes worldwide by 2045 (Saeedi et al., 2019).

The prevention, treatment, and mitigation of diabetes have attracted considerable research resources worldwide. However, despite this effort, problems associated with type 2 diabetes are becoming worse (Saeedi et al., 2019). Type 2 diabetes, accounting for ~90% of diabetes cases, requires effective and long-lasting treatment, due to its chronic and debilitating nature (Wu et al., 2019).

Both type 1 and type 2 diabetes are related to insulin, a hormone produced in pancreatic β cells that signals the removal of glucose from the blood and stimulates the storage of glucose in the form of glycogen (Sullivan and Forbes, 2019). Glycogen is a hyper-branched and randomly branched polymer of glucose, also containing a small but important amount of protein (Meyer et al., 1970; Stapleton et al., 2010). It provides for the storage of energy in a wide range of organisms (from bacteria to mammals) and provides energy to cells on demand and storage of glucose when that molecule is in abundance (Sullivan et al., 2015a; Besford et al., 2015; Wang et al., 2019a). Mammalian glycogen is present in many organs, especially the liver (the highest concentration), and also the skeletal muscle, heart, brain, skin, kidney, and adipose tissue (Markan et al., 2010; Adeva-Andany et al., 2016a). In type 1 diabetes, the pancreatic β cells are destroyed by a chronic autoimmune disorder, severely limiting the amount of insulin produced, and thus resulting in loss of control of the level of blood sugar (Notkins and Lernmark, 2001). In type 2 diabetes, the body develops a resistance to insulin, resulting in a decrease in the amount of glucose that can be removed from the blood and stored as glycogen. The body initially produces higher amounts of insulin to compensate for this resistance; however, this is unsustainable, and eventually, the pancreatic β cells also become damaged, ultimately leading to insufficient insulin action (Stumvoll et al., 2005).

**Figure 1:**
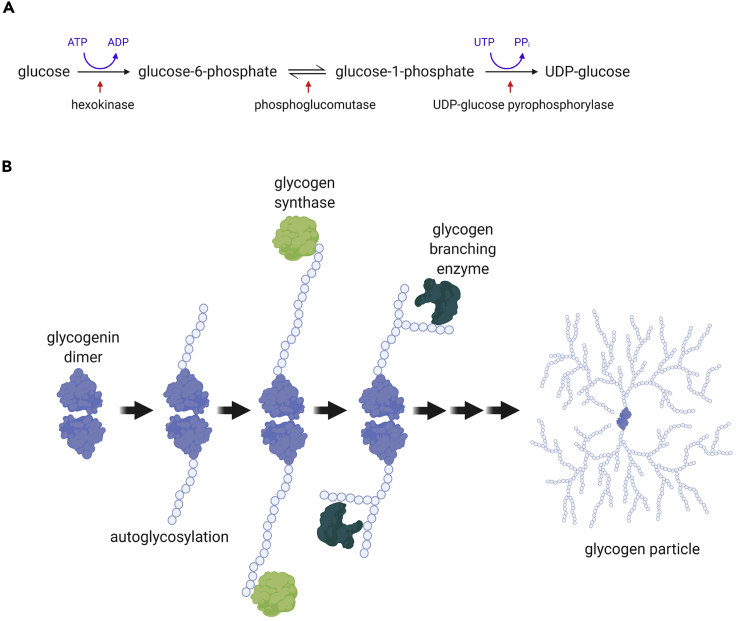
Biosynthesis of glycogen (A) Firstly, glucose is converted to UDP glucose. (B) UDP glucose is then used as the glucose donor for both glycogenin and glycogen synthase, with glycogen branching enzyme creating new branch points.

A summary of the biosynthesis of glycogen is given in Figure 1. The first step involves the conversion of glucose to uridine diphosphate (UDP) glucose (Figure 1A), the substrate for the action of glycogen synthesis enzymes (Roach and Zeeman, 2016). The initiation of a new glycogen molecule involves the autoglycosylation of a glycogenin dimer (Figure 1B). Glycogen synthase (GS) then elongates the dimer and subsequent chains using UDP glucose as the substrate, with glycogen branching enzyme (GBE) creating new branch points (Adeva-Andany et al., 2016b). Of equal importance to glycogenesis is the reverse process, glycogenolysis, whereby glycogen undergoes degradation to glucose. This involves a number of degradative enzymes, especially glycogen phosphorylase, glycogen debranching enzyme, and α-glucosidase (Roach et al., 2012).

**Figure 2:**
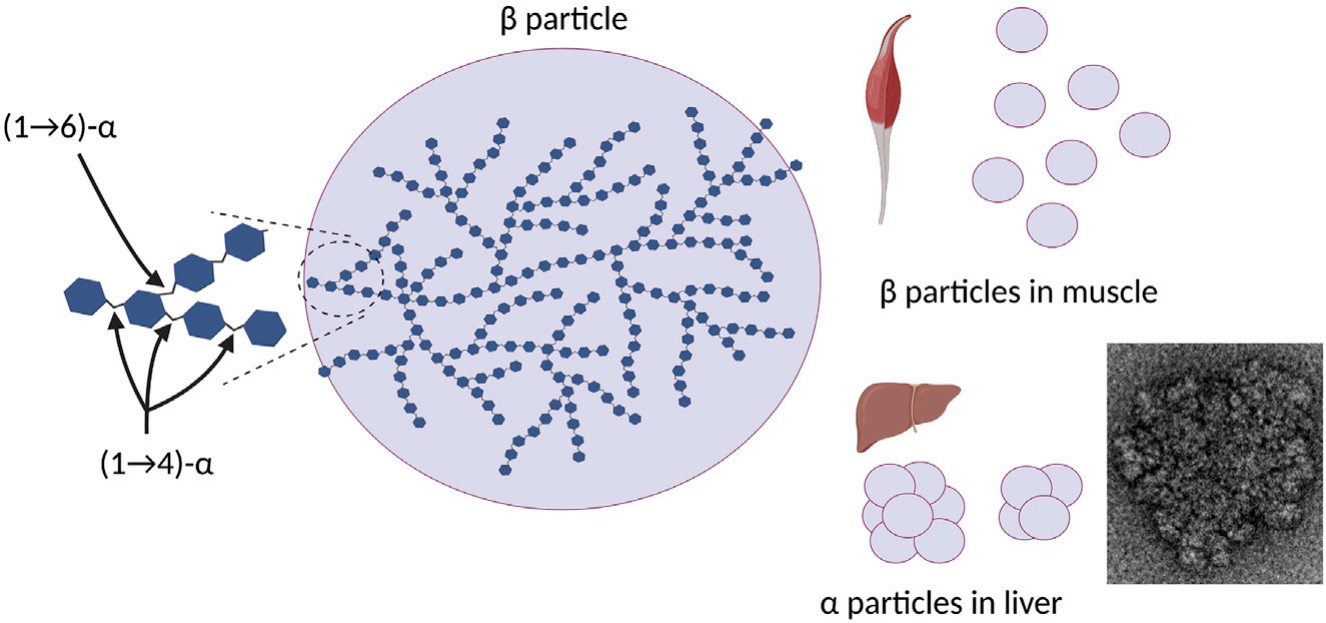
In glycogen, glucose units are held together in a chain via (1→4)-α linkages, with (1→6)-α linkages acting as the branch points, resulting in a glycogen β particle These can be connected to form larger agglomerate α particles via a currently unknown mechanism. A typical TEM micrograph of a glycogen particle (from the authors' laboratory) is displayed, showing small β particles agglomerated into a large α particle.

The glucose units in glycogen are connected via (1→4)-α linear linkages and (1→6)-α branching linkages, resulting in a glycogen molecule (termed β particle). These have a distribution of molecular weights averaging ~10^6^ Da and of sizes with diameters of ~10–30 nm (as seen with transmission electron microscopy, TEM) (Ryu et al., 2009). In the liver, these particles can be connected to form larger α particles, with a granular (agglomerate) appearance under TEM, and with a broad size distribution which has been measured to be up to ~200 nm in diameter, with molecular weights greater than 10^8^ Da (Powell et al., 2015; Melendez et al., 1998; Krisman and Barengo, 1975; Sullivan et al., 2010). These structural features of glycogen are given in Figure 2, as is a typical transmission electron micrograph of an α particle, showing these features.

### The evolutionary driving force for the existence of α and β particles

Liver glycogen acts as a blood glucose buffer and thereby plays an important role in blood glucose homeostasis. Glycogen in other locations (brain, muscle, etc.) functions as a short-term source of glucose for immediate use as energy (Adeva-Andany et al., 2016a). As discussed in detail later, there is a growing body of evidence that diabetes causes key structural changes to diabetic glycogen, potentially decreasing its ability to properly buffer blood glucose concentrations (Deng et al., 2015a; Gilbert and Sullivan, 2014).

It has been pointed out (Wang et al., 2019a) that there is a strong evolutionary driving force for any organism which uses glucose as an energy source and energy reservoir to contain both α and β particles, with the concentrations of each varying with the location in the organ and with the organism's environment and energy need. This is because such particles provide an excellent means of storing and releasing glucose at a rate that is appropriate to the energy need of the organism at that time. In brief, the size of a β particle is fixed by the activities of the various glycogen biosynthesis enzymes and by their sizes and the sizes of the linked glucose monomers (Deng et al., 2015b). These do not vary much between different species, which explains why β particles show only small differences in size distributions in different species and organs (Deng et al., 2016; Sullivan et al., 2012; Wang et al., 2020b). During glycogenesis, there is a need to store glucose quickly, and given the observation (Jiang et al., 2016) that the rates of these processes depend on the surface area of the glycogen particle, glucose storage will be more rapid with smaller particles. By the same token, enzymatic degradation will be slower with larger particles, and slow degradation is needed for controlled glucose release when the organism requires energy. The assembly of β particles into larger α particles would enable this slow glucose release. Hence, evolutionary pressures may mean that both types of particle are present in any organ(ism) with the need for glucose storage, with the ratios of each type of particle, and size distribution of α particles, dependent on the time in the diurnal cycle and on environmental conditions.

### Overview of glycogen structure

The formation of α particles in the liver is a dynamic process across a diurnal cycle, which must be considered when comparing diabetic and non-diabetic glycogen (Sullivan et al., 2014a). Due to the ethical requirements for *in vivo* experiments on humans, mouse models of diabetes have been often used, with only very limited molecular structural data available for human glycogen (e.g. (Deng et al., 2016)). Some structural data on other species such as bacteria, worms, rats, and pigs have also been reported (Hu et al., 2017; Wang et al., 2019a, 2019b, 2020a; Liu et al., 2020).

The highly branched nature of glycogen makes the analysis of its structure technically challenging: a full description of its molecular structure requires an infinite-dimensional infinitely hierarchical distribution function (Gray-Weale and Gilbert, 2009). However, methods have been developed to allow certain aspects to be elucidated. The separation of molecules based on size using size-exclusion chromatography (SEC) (Deng et al., 2015a; Sullivan et al., 2011, 2012, 2014b) has been used in conjunction with various detectors to help determine key structural features of the heterogeneous glycogen populations from mice and other organisms with and without diabetes. The main detectors are differential refractive index (DRI) and multiangle laser light scattering (MALLS) detectors. Both fluorophore-assisted carbohydrate electrophoresis (FACE) and high-performance anion-exchange chromatography with pulsed amperometric detection (HPAEC-PAD) have been used to determine the chain-length distribution (Deng et al., 2015b; Sullivan et al., 2019).

An important consideration when analyzing glycogen structure is the extraction methods that are used. Clearly, when analyzing molecular structure, it is essential to minimize damage to that structure arising from the extraction method and to ensure that the method does not preferentially extract one region of molecular weight over another.

This review first summarizes techniques used to extract glycogen and current methodologies to analyze and describe glycogen's molecular structure. Then, the relationship between glycogen structure and diabetes is discussed in detail and the possible implications for this are addressed, including current results that examine various anti-diabetes drugs and supplements. A better understanding of how total carbohydrate metabolism, including the glucose storage molecule glycogen, is altered in diabetes will provide potentially useful knowledge for the development of more refined and targeted therapeutics.

## Characterization of glycogen molecular structure

### Glycogen extraction techniques and their effect on molecular structure

Before the molecular structure of glycogen can be determined, it must first be extracted from the tissue of interest. Factors to consider when extracting glycogen include the total yield, the purity, and, importantly, the minimization of structural damage and of molecular weight/size bias. Maximizing one of these factors often comes at the expense of the others. For example, a technique that produces high yields often includes more contaminants, decreasing the purity. Many widely used techniques employ harsh conditions, for example, boiling in 30% potassium hydroxide, which can result in relatively high yields with relatively high purity, but also resulting in considerable levels of molecular damage (Stumvoll et al., 2005). Another method that has been used traditionally to extract glycogen employs the use of cold trichloroacetic acid instead of boiling potassium hydroxide; however, it was shown that for liver glycogen this causes significant amounts of degradation (Orrell and Bueding, 1964). This is consistent with more recent studies that show glycogen α particles degrade readily when exposed to acidic conditions (Powell et al., 2015; Deng et al., 2016; Sullivan et al., 2012).

An important consideration is how the extent of structural damage can be assessed. For starch, another complex branched glucose polymer with the same chemical bonds as glycogen, it has been noted (Zhao et al., 2020) that two criteria for minimal structural damage are that there be more large molecules and more long chains, as both of these structural features are the most likely to be damaged. If the objective is to characterize molecular structure, then extraction yield is unimportant, as long as the extraction procedure is not biased in the range of molecular structural features, e.g., more small molecules than large are extracted.

A gentle “cold water” sucrose gradient density extraction method (the “sucrose method”) to extract liver glycogen results in only a small degradation of molecular structure (Ryu et al., 2009; Parker et al., 2007). The method has been shown to extract approximately half of the glycogen and the purity has been measured to be ~50% (Sullivan et al., 2015b). Importantly, the contaminants were found to be small molecules that could be removed using preparative SEC, as has been used (Tan et al., 2016) to yield glycogen of high purity, although not in preparative quantities. The sucrose method however preferentially extracts larger glycogen molecules, with the potential for small glycogen molecules to remain in the supernatant after centrifugation (Geddes and Chow, 1994). It is important to recognize that the glycogen that is subsequently analyzed is therefore not the same to that found in native tissue and that there is a scope for further development of these methods.

A method employed to extract liver glycogen from formalin-fixed tissue has higher yields than the sucrose method (~85%) but with a lower purity (~30%) (Sullivan et al., 2015b). Briefly, this method involves fixing liver tissue in 10% neutral buffered formalin for 48 h at room temperature, followed by homogenization and centrifugation. The glycogen is then purified further using ethanol precipitation. It was shown that the addition of protease to this extracted glycogen led to the detection of significantly more α particles, suggesting that perhaps many of the α particles were aggregated together due to the cross-linking of associated proteins. Again, the contaminating molecules resulting from this method of extraction were much smaller than glycogen and could easily be removed using preparative SEC. A comparison of this method with the sucrose method revealed that less of the small glycogen molecules were lost, and the size distributions were similar enough to indicate that the sucrose method, while imperfect, still extracts a large proportion of small molecules (assuming the formalin method does not, for some reason, preferentially exclude smaller particles) (Sullivan et al., 2015b).

### Molecular size distributions

A useful description of glycogen's complex structure is the distributions of the molecular sizes in a sample. SEC can be used to separate molecules based on their size, specifically the hydrodynamic radius, *R*_h_.

By using a set of standards with known hydrodynamic radii, the elution time of glycogen molecules can be converted to *R*_h_. This conversion depends on the SEC conditions, which can change from day to day and with the particular piece of equipment used, as described in detail elsewhere (Vilaplana and Gilbert, 2010); thus, SEC calibration with the standards needs to be carried out on the day and with the conditions of the sample analysis. The relative concentration (total weight) distribution, *w*(log *R*_h_), of molecules at each size is determined using a differential refractive index (DRI) detector (Vilaplana and Gilbert, 2010). An analogy would be finding the total weight of all trees in a forest of the same height (although in the case of glycogen, “size” refers to the hydrodynamic radius).

The use of a MALLS detector together with a DRI detector allows for the determination of the weight average molecular weight ${\bar{M}}_{W}$ of molecules as a function of their size, *R*_h_, which can be converted to molecular density (defined as ${\bar{M}}_{W}$/^4^/_3_ π R_h_^3^), as has been done for both glycogen and phytoglycogen (Powell et al., 2015). A useful analogy would be to find the average weight of all trees in a forest of the same height; however, it is important to note that ${\bar{M}}_{W}$ is skewed toward larger sizes so it is not identical to the mean weight. The more dense glycogen molecules are, the higher the ${\bar{M}}_{W}$ is for a particular size.

**Figure 3:**
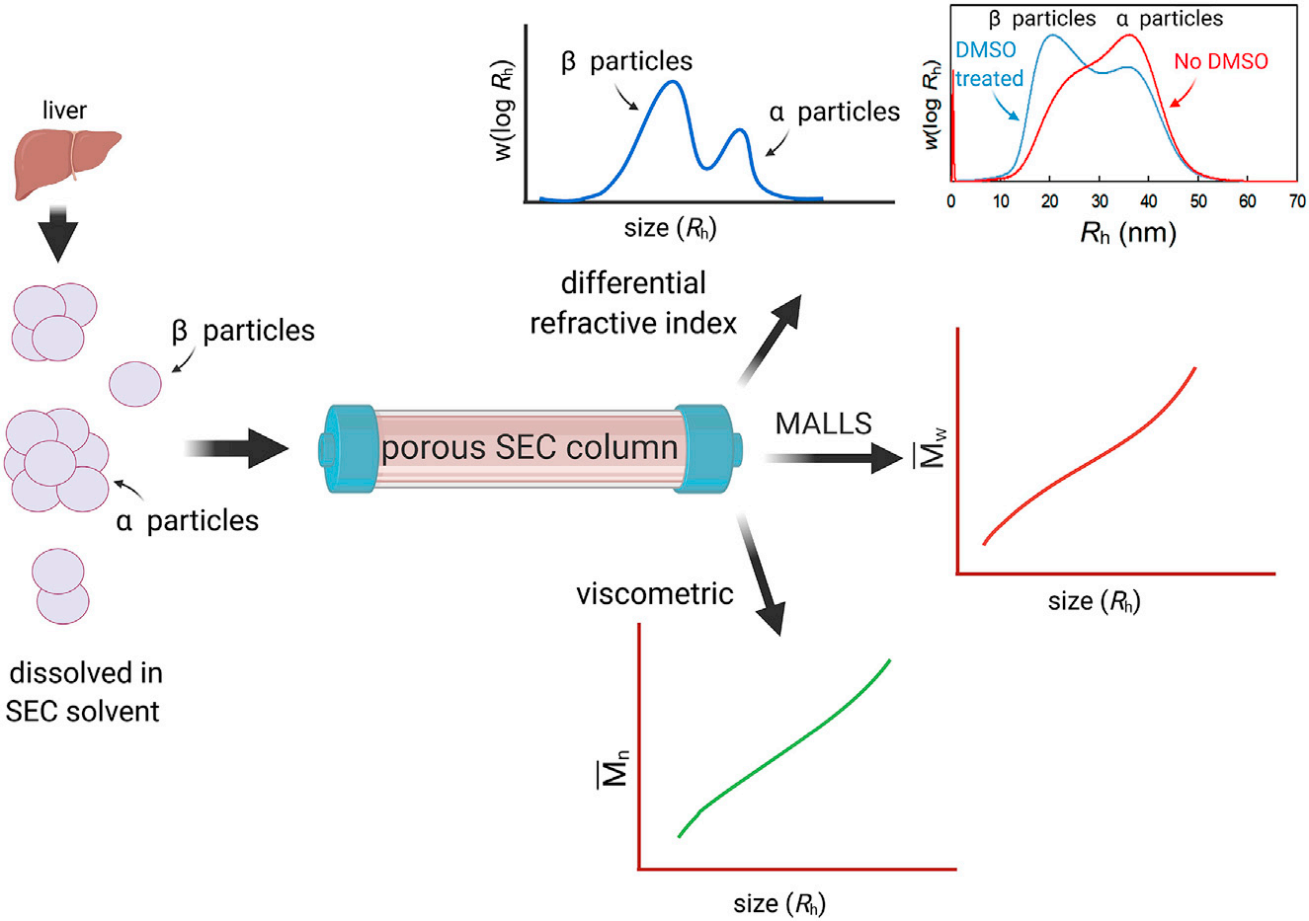
The determination of glycogen's molecular structure Size-exclusion chromatography with multiple detectors allows the determination of the size distributions of the total weight *w*(log *R*_h_), weight average molecular weight ${\bar{M}}_{W}$(log *R*_h_), and number average molecular weight ${\bar{M}}_{n}$(log *R*_h_) of complex branched polymers such as glycogen. The separation parameter for SEC is, by definition, the hydrodynamic radius *R*_h_. Inset top right: SEC distributions for diabetic liver glycogen in water as eluent and after exposure to DMSO in water as eluent (from the authors' laboratory).

The number average molecular weight ${\bar{M}}_{n}$, which is equivalent to the mean weight of trees of each height (or glycogen molecules of each size), can be determined using viscometric and DRI detectors (Gidley et al., 2010). The disparity between ${\bar{M}}_{W}$ and ${\bar{M}}_{n}$ indicates how variable molecular weights of molecules are of each size (or to extend the analogy, weight of trees of each height). Limited multi-detector SEC data for glycogen (Sullivan et al., 2010) and previously published simulations for a generic hyperbranched polymer (Gray-Weale and Gilbert, 2009) indicate that the dispersity Ð =${\bar{M}}_{W}/{\bar{M}}_{n}$, an indicator of the variability in molecular weights at a given size, is very close to unity for complex branched polymers such as glycogen, showing that the measurement of ${\bar{M}}_{n}$ is unnecessary. A summary of how different distributions are measured is given in Figure 3.

Typical *w*(log *R*_h_) distributions for diabetic liver glycogen in water and after exposure to dimethyl sulfoxide (DMSO) are given in Figure 3 (top right inset). One indeed sees that the size of β particles is ~20 nm, with a moderately wide distribution of sizes. This is consistent with TEM measurements (exemplified by the inset in Figure 2). It is recalled that “size” as inferred from SEC, the SEC hydrodynamic radius *R*_h_, is a complicated quantity controlled by the hydrodynamics of SEC elution and is similar to, but not the same, as size inferred from absolute methods such as light scattering and TEM.

Simulations (Zhang et al., 2018; Konkolewicz et al., 2008) show that the average size and size distribution of a β particle is controlled by the various dynamic events of chain growth and branching. A significant influence on these events is hindrance or crowding: both the above mentioned simulations and experiment (Besford et al., 2015) show that the density of a β particle decreases with increasing radius (unlike what had been supposed in many previous publications on glycogen, e.g (Melendez et al., 1998), and in synthetic dendrimers). The simulations show that the average size and size distribution of a β particle is largely controlled by a competition between chain growth (through GS) and chain stoppage by both branching (by GBE) and by hindrance.

### Chain-length distributions

The chain-length distribution (CLD) is the distribution of glucose monomer units in the chains in a sample. It can be equivalently expressed as the weight distribution *w*(log*X*) or the number distribution *N*_de_(*X*), where *X* is the number of monomer units, with *w*(log*X*) = *X*^2^ *N*_de_(*X*) (Clay and Gilbert, 1995). The reason for the notation *N*_de_(*X*) is that it is the number distribution after enzymatic debranching of the whole molecule.

Glycogen chain length has been shown to be important in determining its properties. For example, in many glycogen storage diseases such as Lafora disease and Andersen disease, longer than normal chain lengths cause glycogen molecules to become insoluble, resulting in the formation of pathological “polyglucosan bodies” (Sullivan et al., 2017, 2019).

**Figure 4:**
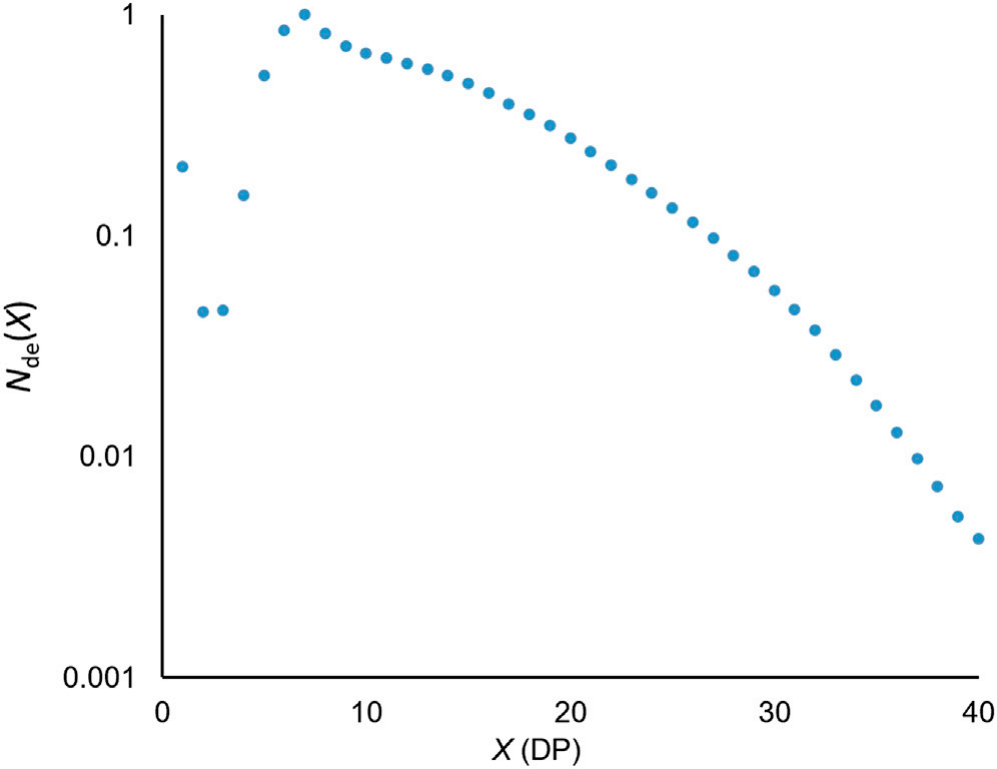
Number CLD from FACE data for debranched human liver glycogen Note that the Y axis is logarithmic. The X axis refers to the number of glucose units in the chain, *X*, also known as the degree of polymerization (DP). This data is from the authors' laboratory.

The first step to measure the distribution of chain lengths in a glycogen sample is to break the sample down into individual chains using isoamylase, which exclusively cleaves (1→6)-α linkages and leaves linear glycan chains. These chains can then be separated based on size using any of SEC, HPAEC-PAD, or FACE (Morell et al., 1998). SEC can separate without restriction on chain length but suffers (i) from band-broadening (and so cannot separate individual chains, which also distorts the actual distribution) and (ii) from uncertainties due to use of the Mark-Houwink equation to relate SEC elution volume (or equivalently elution time) to degree of polymerization (DP) (Cave et al., 2009). FACE and HPAEC-PAD can both separate individual chains and thus both unambiguously identify the amount of chains as a function of their DP. FACE has the advantage over HPAEC-PAD that the signal is independent of chain length, allowing the direct quantification of the relative abundances of chain lengths within a distribution, and also covers the whole range of DPs where there are significant amounts of glycogen. In HPAEC-PAD, longer chains give a more intense signal, making the final distribution skewed toward larger chains (Koch et al., 1998), i.e., it is not quantitative without additional calibration. Figure 4 shows a typical FACE *N*_de_(*X*). Note that the Y axis is logarithmic, for reasons explained elsewhere (Castro et al., 2005).

As yet, data for *N*_de_(*X*) can only be parameterized empirically, as, unlike the equivalent debranched distribution for starch (e.g. (Tao et al., 2019)) (the sister molecule of glycogen), there is no model for the glycogen CLD.

### Liver glycogen molecular structure and diabetes

The first data on molecular size distributions of liver glycogen from *db/db* mice, a mouse model of type 2 diabetes, were interpreted as showing that these diabetic mice were unable to synthesize the larger α particles present in non-diabetic mice, as only β particles were detected (Sullivan et al., 2011). It was later realized and pointed out in a subsequent publication (Deng et al., 2015a) that the SEC eluent used in that first study, DMSO, degrades glycogen α particles from diabetic mouse livers, while the glycogen from non-diabetic livers was less molecularly fragile. A subsequent study using water as the SEC eluent showed that, with an aqueous-based SEC system, diabetic and non-diabetic glycogen have very similar size distributions, but the α particles in diabetic glycogen were readily degraded to β particles in DMSO. It was also found that this phenomenon is not limited to type 2 diabetes, with two different mouse models of type 1 diabetes, non-obese diabetic (NOD), mice and C57BL/6J mice administered with multiple doses of streptozotocin (STZ), also having glycogen α particles that are more susceptible to degradation in DMSO than the non-diabetic controls (Hu et al., 2019). A study of fragility as a function of the time of exposure to DMSO has not yet been carried out. If it were to be found that some particles were non-fragile with exposure to DMSO over very long time periods, then it could be concluded that there are two types of glycogen particles in diabetic liver: fragile and non-fragile. However, this has not yet been tested.

This result raises the following questions, which are yet to be answered. What is causing diabetic α particles to become molecularly fragile? If a phenomenon related to this fragility occurs *in vivo* and as the small particles resulting from this degrade enzymatically to glucose more rapidly (Jiang et al., 2016), does this impact the ability *in vivo* of this fragile glycogen to act as a blood glucose buffer? Could this potentially be involved with the poor blood glucose control seen in diabetes?

To go into more detail, it has been shown *in vitro* that smaller β particles are degraded by glycogen phosphorylase at a faster rate per glucose unit than α particles (Jiang et al., 2016), perhaps due to their greater surface-area-to-volume ratio. From an evolutionary perspective, one reason that α particles form in the liver may be to limit surface area per glucose unit, leading to a more controlled and slower release of glucose during fasting. If fragile α particles in diabetes lead to a higher proportion of β particles *in vivo*, this may be involved in the poorly controlled release of glucose that is a characteristic of diabetes.

Another structural feature that has been observed to be different in diabetic liver glycogen is the chain-length distribution, with *db/db* mice having comparatively longer branch lengths (Hu et al., 2018). Whether this alteration in chain length is linked with α-particle fragility is currently unknown.

### What holds α particle together?

The discovery that α particles in diabetic liver glycogen are molecularly fragile has led to a new focus on the question of what links the smaller β particles together to form an α particle. There are several possible mechanisms for this formation.

Early studies proposed that disulfide binding (Krisman and Barengo, 1975) is responsible for the formation of α particles in the liver, deduced from the observation that 2-mercaptoethanol, a disulfide bond disruptor, degraded the larger particles (Chee and Geddes, 1977). It was then realized that the conditions used in this experiment would result in low pH and that perhaps this could be the cause for particle degradation (Manners, 1991). It was subsequently confirmed that the reagents (including iodoacetamide) in the original 2-mercaptoethanol experiment did indeed result in acidic conditions and that α particles also degraded when exposed to a similar low pH in the absence of 2-mercaptoethanol (Sullivan et al., 2012). This study and subsequent studies with both pig liver (Powell et al., 2015) and human liver (Deng et al., 2016) glycogen demonstrated that the link between α particles in these organs and organisms is much more susceptible to acid hydrolysis than are the normal glycosidic linkages found in glycogen. This showed that the link was not simply a long glucose polymer chain, as had been hypothesized earlier (Matsuda and Hata, 1985), because the bonding in such a chain is not susceptible to mild acid hydrolysis.

It was also proposed that a protein “glue” was a possible candidate because degradation of α particles into smaller β particles was also seen when glycogen was exposed to formalin and heat (Sullivan et al., 2015b). Formalin reacts quickly with proteins but has been shown to have relatively little effect on carbohydrates (Eltoum et al., 2001). The breakdown of α particles when exposed to formalin is consistent with, but does not prove, a protein “glue” binding the β particles into α particles in these organs and organisms and appears inconsistent with the linkages being simply long glucose polymer chains. It should be noted that protease treatment does not break apart α particles (Sullivan et al., 2015b), which appears contradictory to the hypothesis that the linkage holding them together is proteinaceous; however, it is conceivable that the protease is unable to penetrate into the interior of α particles due to a barrier of dense glycogen chains.

A proteomics study performed on fractionated liver glycogen enriched in either β or α particles found that the glycogen-initiating protein, glycogenin, was the most likely candidate for this protein “glue” (Tan et al., 2018). Not only was there a significant increase in the amount of glycogenin protein enriched in α-particles compared to ones enriched in β particles but there was no evidence of other candidate proteins. This does not prove that glycogenin is the “glue” linking β particles together in the liver, but it is consistent with this hypothesis. It is important to find additional tests that could support or disprove this suggestion.

### Diurnal changes in liver glycogen structure

It has been shown using mouse models that the molecular size distributions of liver glycogen are dependent on the time in the mouse's natural feeding cycle at which the animals are killed (Sullivan et al., 2014a). In a natural-light diurnal cycle, mice stop eating at about 8 am. It has been shown in mice that at midnight, 4 am, and 8 am, glycogen α particles are molecularly fragile in both healthy and diabetic (*db/db*) mice, whereas at noon, 4 pm, and 8 pm, it is only diabetic mouse liver glycogen that degrades when dissolved in DMSO, with the non-diabetic liver glycogen being stable in DMSO (Hu et al., 2018). It was suggested in this paper that in non-diabetic mice, glycogen particles become more stable as the day progresses, a process that is lacking in diabetic mice. Another observation in that study was that as the α particles became more stable, their molecular density decreased, with diabetic mice having α particles of a higher density than non-diabetic mice at 8 am, 12 noon, 4 pm, and 8 pm. The physiological significance of this difference is currently unknown.

### Molecular fragility of diabetic α particles reversed with some antidiabetic treatments

The discovery that glycogen α particles were molecularly fragile in both type 1 (Hu et al., 2019) and type 2 (Hu et al., 2018) diabetic mice suggested that a cause of this molecular abnormality may be uncontrolled blood glucose levels. To test this, *db/db* mice were treated with four different active ingredients from traditional Chinese medicines that have been shown to have antidiabetic properties (Li et al., 2019). Of the four preparations, three preparations (astragalus polysaccharide, berberine, and pueraria flavonoid) significantly lowered blood glucose levels, while one (panaxnoto ginseng saponins) had no significant effect. The three preparations that lowered blood glucose levels also reversed the fragility of the fragile α particle phenotype, while the other, the saponin preparation, that had no effect on blood glucose also had no effect on glycogen structure. This supports the hypothesis that blood glucose levels are correlated to fragile liver glycogen particles; however, further studies are needed to corroborate this initial study and elucidate the mechanism involved in this link. Whether or not fragile liver glycogen particles exacerbate poor blood glucose control, as opposed to being simply caused by it, is a crucial question in determining whether this research may lead to a new therapeutic target.

### Updated model for diabetic glycogen structural differences

**Figure 5:**
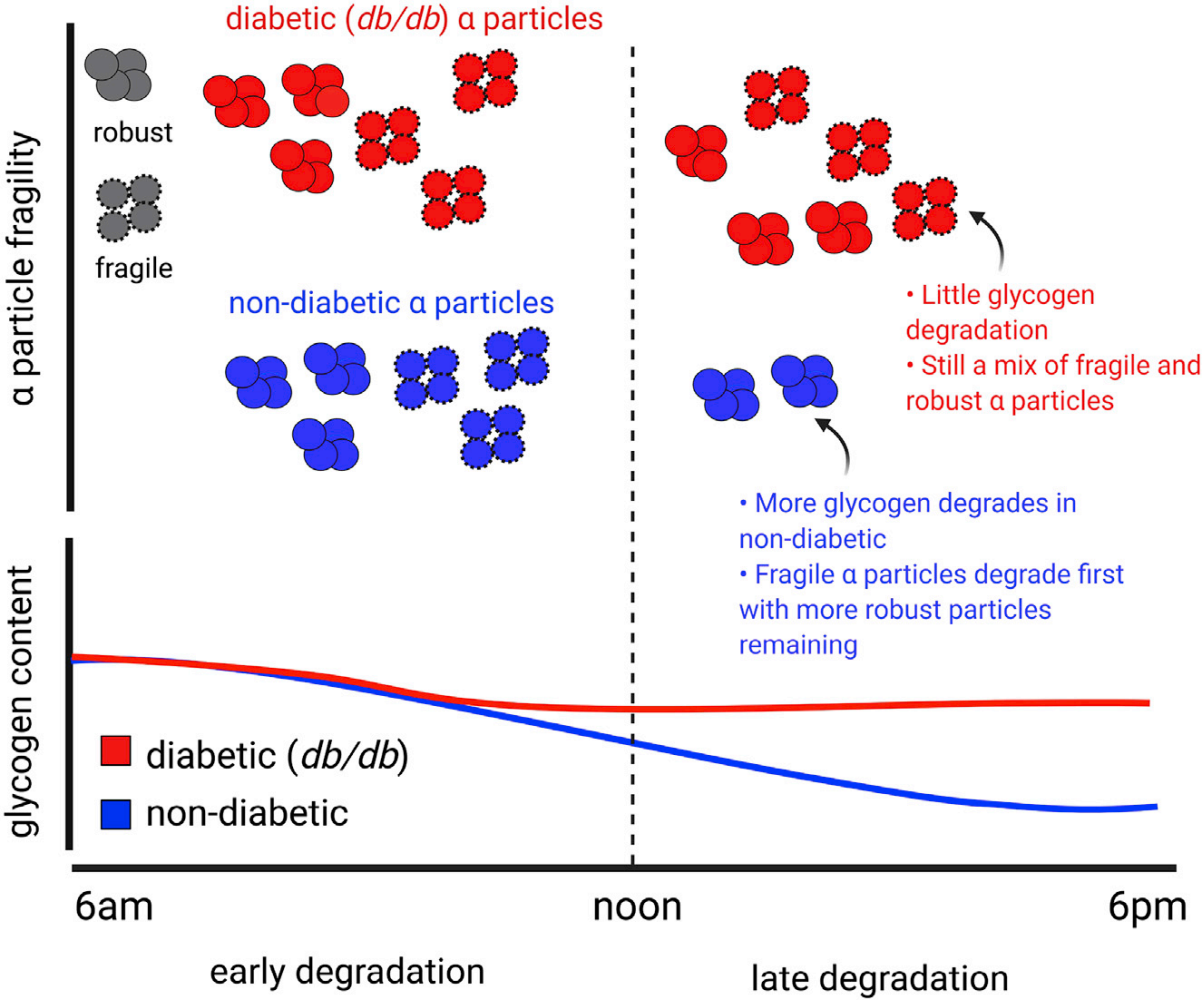
An updated model explaining differences in diabetic (*db/db*) and non-diabetic glycogen structure, hypothesizing two types of glycogen α particles

An updated model that is consistent with the differences observed between diabetic and non-diabetic α particles is presented in Figure 5. While more data are needed to validate this model, it may be useful to present it as a possible alternative to models previously published. This model differs from previous ones inasmuch as that it takes into account that there may be two types of glycogen α particles in diabetes: potentially fragile and robust to fragility. This model suggests that when glycogen levels are high, both non-diabetic and diabetic liver glycogen contains a mixture of both potentially fragile and robust particles (the terms robust and fragile refer to degradation upon exposure to DMSO). This is consistent with all extant data on glycogen size distributions (obtained by non-degradative extraction methods) showing that glycogen samples before and after DMSO treatment always have some residual α particles (Deng et al., 2015a; Hu et al., 2018, 2019; Li et al., 2019).

Instead of the previous model wherein non-diabetic glycogen would become more robust as the day progresses (from 8 am to 4 pm), this model suggests that the data are consistent with a situation where fragile particles preferentially degrade.

When glycogen content is high (for mice, at beginning of the light cycle), diabetic and non-diabetic glycogen both contain a mixture of robust and DMSO-fragile α particles. The diabetic glycogen content does not change much through the day, so this distribution of particles remains constant. The non-diabetic glycogen, however, is degraded. This model suggests that the fragile particles preferentially degrade, leading to an enrichment of robust particles.

In this model, as the glycogen content decreases and fragile particles preferentially degrade, the proportion of robust particles increases. In diabetic (*db/db*) mice, however, it has been shown that the glycogen content remains high throughout the daylight hours (presumably due to their excessive eating throughout this time), while control mice stop eating (Roesler et al., 1985). Therefore, this process of preferential degradation of the fragile particles is less pronounced than that in non-diabetic mice, with the distribution of particles being similar at the beginning and end of daylight hours.

If this updated model is applicable, there should be a correlation between glycogen content and the relative number of fragile particles. A higher proportion of dense/fragile particles were observed (Hu et al., 2019) in two models for type 1 diabetes (NOD and STZ induced), but unfortunately, the glycogen content was not reported. Repeating this experiment would provide evidence supporting or refuting the present hypothesis.

It should be noted that this proposed model suggests that the enrichment of fragile particles is a side effect of poor blood glucose control. Importantly, while not the primary cause of poor blood glucose control, given the finding that β particles degrade faster than larger α particles (Jiang et al., 2016), this may still lead to a positive feedback loop where diabetic glycogen is more susceptible to a fast degradation, potentially exacerbating poor blood glucose control.

## Conclusions and future perspectives

### Implications for potential therapeutics

The manipulation of glycogen metabolism so as to help control blood glucose levels in diabetes is well explored. For example, slowing down glycogen degradation with glycogen phosphorylase inhibitors has been shown to exhibit beneficial effects on blood glucose control in models of type 2 diabetes (Docsa et al., 2011; Martin et al., 1998; Hoover et al., 1998). The targeting of glycogen synthase kinase-3 (GSK-3) to help control blood glucose in diabetic mice has also shown some promise, with an inhibition of GSK-3 resulting in increased glycogen synthase activity and more glycogen produced (Macaulay and Woodgett, 2008).

If the differences in molecular structure between diabetic and non-diabetic glycogen particles are shown to be physiologically relevant to glycogen degradation and therefore to blood glucose control, this feature of carbohydrate metabolism would be a potential therapeutic target (Sullivan et al., 2015a). An important consideration is that this therapeutic would not be limited to trying to make diabetic glycogen resemble non-diabetic glycogen. Having a thorough understanding of the structure/property relationships of glycogen could allow favorable alterations targeted directly to the problem of high blood glucose levels. For example, a therapeutic target could include increasing α-particle size and stability in diabetic livers through administration of appropriate agonists or antagonists at various parts of the glycogen cycle, slowing down glycogen degradation. This specific possibility is contingent on determining what holds α particles together. If glycogenin is indeed the protein “glue”, perhaps increasing expression of this protein would lead to larger and more stable α particles. Another possibility as a therapeutic target is to use drugs which modify the activities of enzymes involved in glycogenesis and glycogenolysis, thereby controlling the rates of these two processes. A greater understanding of α particle formation is needed before any such therapeutic becomes possible.

### Final thoughts

The development of improved drugs for diabetes management is a priority for global public health. The observed molecular structural differences in healthy and diabetic glycogen present an interesting possibility for conceiving new therapeutic targets aimed at improving blood glucose control. For example, if we had a better understanding of what causes diabetic liver glycogen to be more molecularly fragile, we could begin to attempt designing therapies to reverse this phenotype. One possible way would be to increase the stability of diabetic α particles by increasing the expression of the hypothesized protein “glue”. While an intriguing possibility, further research is needed to gain a clearer understanding of the cause of these differences and the physiological relevance.
